# Spirituality and Cognition in Black Older Adults: Examining Changes in Church Activity

**DOI:** 10.1002/alz.092532

**Published:** 2025-01-09

**Authors:** Katherine Carroll Britt, Fanghong Dong, Lauren M Massimo, Jill B Hamilton, Nancy A Hodgson, Shana D. Stites, Dawn Mechanic‐Hamilton

**Affiliations:** ^1^ the University of Pennsylvania, Philadelphia, PA USA; ^2^ Washington University in St. Louis, St. Louis, MO USA; ^3^ University of Pennsylvania, Philadelphia, PA USA; ^4^ Emory University, Nell Hodgson Woodruff School of Nursing, Atlanta, GA USA; ^5^ Department of Psychiatry, Perelman School of Medicine, University of Pennsylvania, Philadelphia, PA USA

## Abstract

**Background:**

Historically underrepresented populations experience a disproportionate burden of Alzheimer’s disease and Alzheimer’s disease related dementias (AD/ADRD) compared to White populations. As a salient resource for coping in Black communities, spiritual and religious practice may support better cognitive health, but it is unknown if changes in these practices are related to cognitive decline.

**Methods:**

We analyzed secondary data of cognitively normal (via Consensus Conference at enrollment) older adults in the U.S. (*N* = 158) from The University of Pennsylvania Alzheimer’s Disease Research Center (ADRC) Aging Brain Cohort (ABC) in 2021‐2022 to examine associations between change in spiritual and religious practice (i.e., major change in church activity) with cognition, separately between racial groups (Black (n = 44), White (n = 114)). Self‐reported age, sex, education, and number of friends interacted with monthly were entered as control variables in multivariable regression analysis.

**Results:**

Cognitively normal participants had a mean age of 74.4(6.54) years, were 64.6% female, and reported 16.7 (2.40) mean years of education. Significant associations were found for Black but not White older adults between major change in church activity (measured with one item: major change in church activity during the previous year (i.e. a lot more or less than usual)) with lower cognition (measured with Global Clinical Dementia Score) (*β* = 0.19, 95%CI [0.04, 0.34], *p*<0.05) controlling for age, social interaction, sex, and education.

**Conclusion:**

A reported change in church activity over the last year was associated with a lower rating of cognitive and functional status among Black but not White older adults. As some individuals may progress, an early indicator of cognitive or functional change may be reported changes in church activity for older Black adults, for which spirituality is a cultural resource that may support cognitive health. Longitudinal studies are needed to further assess temporal associations between change in church activity and cognition in Black communities; maintaining church activity may be important in supporting cognitive and functional stability. The absence of significant findings in older White adults may be influenced by cultural differences that warrant further investigation and consideration in future research.

